# Erratum for Huang et al., “Insights into the regulatory mechanisms and application prospects of the transcription factor Cra”

**DOI:** 10.1128/aem.00047-25

**Published:** 2025-01-31

**Authors:** Ying Huang, Kai-Zhi Jia, Wei Zhao, Li-Wen Zhu

## ERRATUM

Volume 90, no. 11, e01228-24, 2024, https://doi.org/10.1128/aem.01228-24. Page 12: Affiliation 2 should read “State Key Laboratory of Microbial Technology, Shandong University, Qingdao, China.”

